# Autoimmune Hepatitis: Diagnostic Dilemma in the Setting of Suspected Iron Overload

**DOI:** 10.1155/2013/872987

**Published:** 2013-08-19

**Authors:** In Chul An, Ashish K. Tiwari, Srujan Ameda, Heather S. Laird-Fick

**Affiliations:** Department of Medicine, Clinical Center, Michigan State University, 788 Service Road, East Lansing, MI 48824, USA

## Abstract

Autoimmune hepatitis (AIH) is an inflammatory condition of the liver that has a multitude of clinical presentations from chronic hepatitis to acute fulminant hepatitis. AIH diagnosis is typically suspected after ruling out other causes of hepatitis (such as vial hepatitis, hemochromatosis, Wilson's disease, and primary biliary cirrhosis) through serological tests and by findings of high titers of certain autoantibodies (ANA and anti-SMA for type 1 AIH and anti-LKM-1 for type 2 AIH). AIH like most inflammatory conditions is associated with increased ferritin levels (acute-phase reactant) but typically near-normal transferrin saturation. The presence of excessive ferritin level in absence of high-transferrin saturation helps differentiate secondary iron overload from hemochromatosis where transferrin saturation is typically high. We herein describe a case of AIH that presented with high ferritin levels and transferrin saturation suggesting a diagnosis of hemochromatosis and needed arduous work-up to arrive at accurate diagnosis of AIH.

## 1. Introduction

Autoimmune hepatitis (AIH) is an inflammatory condition of the liver that has a multitude of clinical presentations from chronic hepatitis to acute fulminant hepatitis [1, 2]. Typical symptoms are nonspecific and can include fatigue, jaundice, nausea, and abdominal pain. It is a relatively rare disease affecting women more than men (3.6 : 1 ration) with mean incidence of 1-2 per 100,000 persons per year and prevalence of 11–17 per 100,000 persons [1, 2]. The diagnosis is based on histological findings and presence of one or more specific autoantibodies (ANA and anti-SMA for type 1 and anti-LKM-1 for type 2). Other chronic liver diseases that could often have similar presentations include viral hepatitis, primary biliary cirrhosis (PBC), primary sclerosing cholangitis (PSC), and hereditary liver disorders such as alpha-1 antitrypsin deficiency, Wilson's disease, and hemochromatosis. Although serological tests to detect antibodies are useful initial tests, they could be indeterminate and are not very specific. Liver biopsy is usually needed to differentiate AIH from other causes and needs a careful interpretation by expert pathologist to reach a final diagnosis.

Inflammatory conditions are often associated with elevated ferritin level because the latter is an acute-phase reactant [4]. In case of liver, elevated ferritin levels are most commonly related to alcohol abuse, chronic viral hepatitis, and nonalcoholic fatty liver disease (NAFLD) [3]. However, serum transferrin saturation in such cases is typically within normal range or only slightly elevated, despite elevated serum ferritin [4]. Thus, in appropriate clinical setting, the presence of very high transferrin saturation (>45%) is generally suggestive of primary hemochromatosis and serves as threshold to order HFE-gene testing to diagnose hemochromatosis. We herein present a challenging case of AIH (type 1) with unusually high transferrin saturation (89%) that required arduous evaluation to arrive at final diagnosis.

## 2. Case Presentation

A 53-year-old African American female university professor with history of hypothyroidism due to Hashimoto's thyroiditis presented to Internal Medicine Clinic with jaundice, malaise, and dark urine for past three weeks. She travelled to Africa annually, and her most recent travel was four months prior to presentation. She categorically denied any prior liver or kidney disease, intravenous drug use, previous blood transfusions, alcohol use, and high-risk sexual behavior.

**Figure 1:**
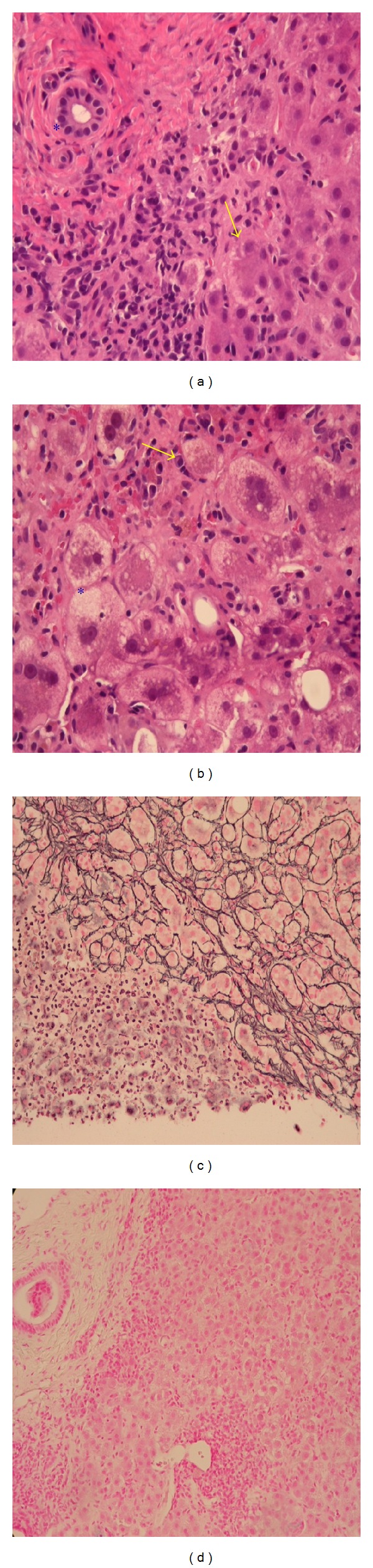
(a) H&E stain showing bile duct (*) and hepatocytes (arrow) with extensive infiltrates (interface hepatitis). (b) H&E stain showing hepatocyte injury/ballooning (*) and plasma cells (arrow). (c) Reticulin stain showing reticulin crowding indicating extensive fibrosis and loss of hepatocytes. (d) Iron stain showing no evidence of excessive hepatic iron deposition.

Initial laboratory evaluation revealed ALT 1,035 IU/L, AST 738 IU/L, total bilirubin 10 mg/dL (direct fraction 8 mg/dL), and alkaline phosphatase 135 IU/L. Imaging studies including abdominal ultrasound and magnetic resonance cholangiopancreatography (MRCP) were normal without evidence of intra- or extrahepatic cholestasis. Additional work-up including viral hepatitis panel, acetaminophen level, ceruloplasmin, alpha-1 anti-trypsin, ANA, anti-SMA, and antimitochondrial antibodies was unyielding. However, iron studies showed ferritin 1570 ng/mL, total iron 258 mcg/dL, and transferrin saturation 89%. A possibility of primary hemochromatosis was strongly considered, and additional diagnostic tests (liver biopsy and genetic studies for HFE protein) were ordered. Interestingly, genetic studies for HFE gene did not support the diagnosis of hemochromatosis, and liver biopsy in fact revealed interface hepatitis with numerous plasma cells (Figure 1), consistent with autoimmune hepatitis. A repeat serology performed after liver biopsy report was strongly positive for anti-SMA (1 : 2560). Based upon these results, a diagnosis of autoimmune hepatitis (type 1) was made. The patient was promptly started on prednisone and azathioprine, and eventually attained normalization LFTs and resolution of symptoms.

## 3. Discussion

Many chronic liver diseases such as nonalcoholic fatty liver disease (NAFLD), chronic hepatitides B and C, PBC, and alpha-1 antitrypsin deficiency are associated with mild iron overload with near-normal transferrin saturation rarely exceeding 45% (the threshold fasting transferrin saturation to initiate further hemochromatosis work-up) [3, 4]. However, in the case presented herein, the transferrin saturation was 89%, strongly (but erroneously) suggesting the diagnosis of hemochromatosis. However, the absence of other end-organ damage (kidney, heart, and pancreas) and a negative HFE-genetic test indicated an alternate diagnosis. Liver biopsy showing the presence of interface hepatitis with lymphocyte predominance and abundance of plasma cells confirmed the diagnosis of AIH in this patient, and iron stains not demonstrating any hepatocyte predominant iron deposition ruled out primary hemochromatosis.

The exact mechanism for such high transferrin saturation is unclear. Dysregulation of iron homeostasis in enterocytes and release of iron from hepatocytes following cell damage and fibrosis could be possible mechanisms. However, the genetics behind the secondary iron overload remains poorly defined. While the homozygous point mutations of HFE-gene (C282Y, H63D, and S65C) are rare and present in about 85% of patients with hemochromatosis, the heterozygous mutations are much more common affecting approximately 6–10% population in Western Europe [5]. Interestingly, the presence of heterozygous C282Y mutation is associated with increased iron liver concentration in chronic liver disease patients. In a study performed in Germany of 531 patients, 17.2% of patients diagnosed with AIH had heterozygous C282Y mutation [5]. Moreover, there are other point mutations including H63D and non-HFE gene mutations such as hemojuvelin (HJV), hepcidin (HAMP), transferrin receptor 2 (TfR2), and ferroportin (SLC40A1) that are shown to cause milder degree of iron overload [6]. However, these mutations seem to have variable penetrance with only one in ten individuals with heterozygous mutations having elevated serum iron profile. In the case presented herein, HFE genetic test was negative. Because this genetic test screens only for C282Y, H63D, and S65C mutations in the HFE gene, it is possible that our patient had a genetic predisposition to iron overload through non-HFE gene mutation mechanisms.

In the present case, female gender of the patient was a risk factor for autoimmune hepatitis (female : male ratio, 3.6 : 1) and protective against a diagnosis of hemochromatosis (female : male ratio, 1 : 2). Additionally, a history of Hashimoto's thyroiditis was another association suggestive of an immune-mediated etiology for hepatitis in this patient. 

## 4. Conclusion

The presence of very high transferrin saturation can pose a diagnostic dilemma in the setting of new-onset hepatitis. Diagnosing AIH can become incredibly difficult without liver biopsy in such cases. A misdiagnosis could lead to institution of incorrect treatments and have potentially lethal implications for the patient. Our case emphasizes the importance of liver biopsy in helping determine the etiology of new-onset hepatitis where initial work-up has been inconclusive and dangers of relying on transferrin saturation for presumptive diagnosis of hemochromatosis pending HFE-gene testing.
